# Hospital Meetings, &c.

**Published:** 1901-07-20

**Authors:** 


### HOSPITAL MEETINGS, &c.

### ROYAL MEDICAL BENEVOLENT COLLEGE, EPSOM.

LORD ROSEBERY ON THE MEDICAL PROFESSION.

The EARL OF ROSEBERY presided at the twenty-eighth anniversary festival of the Royal Medical Benevolent College, Epsom, held on the 9th instant at the Hotel Cecil, when some 350 guests sat down to dinner, among them being many of the leading lights of the medical profesion.

In giving the toast of "The King" the CHAIRMAN mentioned that His Majesty had been pleased to consent to succeed her late Majesty as Patron of the college, thereby adding one more to the many proofs he had already given of his interest in the medical profession.

Proposing the toast of the evening, "Success to the Royal Medical Benevolent College," Lord ROSEBERY said: I have presided at many dinners, but I do not know that I have ever presided at a more alarming one than this. In the first place one's diet becomes a subject of anxious consideration when dining is carried on under the eyes of so many scores of competent critics. (Laughter.) In the next place I cannot but be aware that probably everyone in this room is better informed on the subject on which I am to address you than I am myself. That is a more familiar feeling to me than the other. (Laughter.) Neither of them is perfectly agreeable. Regarding the subject that brings us together to-night, the Royal Medical Benevolent College, you all of you, as I have already said, know more than I do. The only complaint I have to make is that the adjectives in the title are superabundant, and I think it would be better if we dropped them altogether. There may be technicalities which prevent you dropping the adjectives, but I think it would be better if you called it Epsom College, with "Royal Medical Foundation" in brackets beneath. So long a name is the reverse of advantageous—even the boys themselves dislike it. Epsom College is about the same length as Eton College, and I see no reason why Epsom College should not rise to the same height of distinction as any among the secondary schools of the Kingdom. You have the statistics of the college before you so that I need not go into detail, but the facts are these: There is the foundation—I leave out the pensioners for I don't quite know how they became connected with the college—there are 50 exhibition scholarships which have to be maintained, and there are the other children of medical men, and of those who are not doctors, to be provided for. The expenditure represents about £6,000 a year, and the fixed income not being one-third of that amount it is quite obvious that considerable sums of money are needed from outside sources. Indeed, when I think of the task which the treasurer has to face, and the manner in which he does it, I feel that if anything were to happen to Sir Michael Hicks-Beach in the present condition of the national finances, I should wish our treasurer to succeed him. ("Hear hear" and laughter.) These are points, however, with which I have no practical acquaintance, but I can tell you something of the school itself, because I am a near neighbour. (Hear, hear.) I take the greatest interest in this school, and am privileged to attend their matches and even their speech-days. I am privileged to know some of the lads as they pass through the school, and nowhere will you see finer lads than those of Epsom College. (Hear, hear.) I do not believe that any who are conversant with the school curriculum and with the position which the boys hold could refrain from giving it their enthusiastic support. (Hear, hear.) But that is personal evidence. Considering the nature and conditions of the college, and how closely it is allied to the medical profession, we have some right to ask for a larger snpport from the public and from the profession. From the profession first and also from those who are benefited by it. (Hear, hear.) I think we are a little apt to imitate the devil, in the characteristic couplet of which we know the refrain: "The devil was sick, the devil a saint would be"—and then the next line points out, in language almost profane, that when the devil got better he changed his mind. (Laughter.) I do not wish to associate us poor mortals with so eminent or so sinister a person, nor do I take the exact line of the couplet, but I do feel that when the human being is well he is apt to forget to whom it is he owes it that he is well. There are many brilliant exceptions to that rule, but it is perhaps not unnatural to human nature that when it has recovered from a period of sickness and depression it should try to shake off the associations of that period—even the best associations of all, the doctor and the nurse. But when it is brought to thei minds I do not think those who have been ill and have owed so much to those who have cured them, I do not think that on an occasion of this kind they can forget it nor how we can show our gratitude, indirectly, by helping this great institution. But that after all is a personal view—the personal gratitude of the patient to the profession. Do we not all—all humanity—owe a debt we cannot estimate or limit to the medical profession? I suppose in an audience like this I am sure of ready concurrence in the opinion that that profession is one of, if not the noblest, of all the professions. It must be associated with much of pain, where the physician is baffled by the inexorable forces of nature: all professions, all callings, have their failures. But of the medical profession alone, perhaps of all secular professions, it may be said of a man setting out on his day's work, that he knows he can have no object but a beneficent object—to relieve and assuage pain, and to combat, though perhaps with ill success, the chill Angel of Death. That is a consideration I think which comprehends all humanity in a debt of gratitude to the medical profession. But on the other hand if the profession is equal to its duty, whether on the battlefields of South Africa or in the no less arduous task of combating disease and evil conditions of life, surely, if they are ready, as we know they are to perform their duty under all conditions, it is obvious that they pursue that task only at the risk of their own lives. Of course, it is very easy in an assembly like this to fancy that the medical profession is nothing but a brilliant and a lucrative one, but when you come to think that there are some two thousand medical graduates turned out every year; that there are thirty thousand men of medical standing in Great Britain, or outside Great Britain momentarily residing abroad, one cannot but feel that the number of blanks in that profession is out of all proportion to the number of prizes. We have the princes and the prizemen at this table to-night, but these are only a small percentage of the great numbers in country places, and practitioners in towns, who are at the beck and call of disease and pain by day and night, and who must face at every hour in all climates the call of suffering and want. These men when they die, die as surely in the service of their country as those who fall charging batteries. (Cheers.) And many of them leave behind wives they cannot maintain, children they cannot educate. It is for those children we appeal to-night. There was one medical student—the worst of physicians, I believe, but the most famous of medical students—who, in the eighteenth century, lay dying not far from here; and as he lay dying they gathered round him and asked him "Is your mind at ease?" I will quote his answer in the words of Lord Macaulay which I have never forgotten since I read them, "No it is not!" he said, and they were the last recorded words of Oliver Goldsmith. They are remembered because he was famous, but they are the words of thousands belonging to that great profession to which so many here belong, and it is for these I ask you to-night to come to the assistance of Epsom College with the assurance that whatever you give will be well and worthily expended, and that you will have done no better deed than helping to maintain this noble institution. (Loud cheers.)

Dr. Constantine Holman, the treasurer, responded to the toast. He said he thought the public did not do its duty toward that institution to the extent it ought and that more help should be given by those who benefited by the skill and devotion of the profession. With regard to the alteration in the title advocated by Lord Rosebery, they had taken power under their last Act of Parliament to wipe away the adjectives "Medical and Benevolent." If they would give the Council £50,000 the benevolent side of the institution would be secure in the future.

Mr. John Lumsden Propert proposed the health of the Chairman. Not only was he known to them as a statesman, an orator, and a sportsman, but as their kind and sympathetic friend. Not only was he their chairman that night but he was president of the college, and in that capacity was no mere ornamental figurehead. He could tell of many instances of his kind forethought and consideration which had endeared him to all who had the welfare of the college at heart.

The toast was drunk with musical honours, after which the Treasurer announced, amid cheers, that in consequence of the Chairman's address a friend had promised to send a cheque for a thousand guineas in the morning.

Returning thanks for the toast the Chairman said: I am quite sure no speech I could make could be so effectual as the few words just uttered by your treasurer. (Hear, hear.) That is the sort of friend I should like to have, and I am glad to feel that Epsom College has such a friend. (Hear, hear.) I hear Dr. Farquharson, an eminent politician, cheering me, and it fills me at this moment with a clammy feeling that it is just now dangerous to attend a public dinner. (Laughter.) But I hope Dr. Farquharson may not suffer in reputation, and that no dinner of reprisal may follow upon this. (Laughter.) Mr. Propert said he might have enlarged on many sides of my character, but I am rather surprised under present circumstances that he should have selected the sporting side for his commendation. But since he has enlarged on this topic, and it might be the cause of suspicion as to the character of the college over which I preside, and might check enthusiastic friends of the treasurer from sending in anonymous donations, I think it right to say that I understand, from inquiry made on the spot, that during Epsom races the college is kept to bounds with a strictness that nothing is allowed to alter. (Laughter.) Still, I should like to mention that in another branch of our national sports we have two brothers of the name of Heygate who make hundreds of runs with the greatest regularity. (Cheers.) To select this moment for extolling me as a sportsman is not very happy, because I have become literally a one-horse man. (Laughter.) I am not even sure I have one horse. (Laughter.) However that may be, I have collected the Olympian dust in my day; I do not repent it, and I hope I may collect some more. (Cheers.) Well, Lord Beaconsfield used to say that on a Sunday afternoon the egotism of a landed proprietor knew no limits—(laughter)—but on occasions of this sort even the egotism of the chairman must know some limits, and, with the sincerest thanks to you for the way in which you have received this toast, I resume my seat. (Cheers.)

It was then announced by the secretary that, including the 1,000 guineas just mentioned, the subscription lists that evening totalled £4,127, after which the company separated.

### ROYAL EYE HOSPITAL.

The festival dinner of the Royal (South London) Eye Hospital, Southwark, was held at the Hôtel Métropole on Monday, the 8th instant, Sir Blundell Maple presiding. After the Loyal toasts had been duly honoured, Mr. W. M. Chinnery, the President of the hospital, proposed "The Houses of Parliament," and this was responded to by Mr. James Bailey, M.P. Mr. Frank M. Ponder proposed "The Imperial Forces," which was acknowledged by Captain Middleton, R.N., Major-General Sir Francis de Winton, and Captain Houghton-Gaskell for the Navy, the Army, and the Auxiliary Forces respectively.

The Chairman then proposed the toast of the evening. "Success to the Royal Eye Hospital." They were all assembled, he said, to try to do some good for that beneficent institution, and it was his especial province to ask everyone to open their hearts and their purses to the fullest possible extent. They would agree with him that nothing worse could be imagined as happening to anyone than the loss of his eyesight. In this vast metropolis there were many who would be unable to fight the battle of life if it were not for the aid afforded them by this hospital. He wished all there could go as he had gone the other day and inspect and examine the working of this admirably arranged institution; to see how beautifully it was all planned and carried out for the comfort and convenience and well-being of the patients; and to witness the work of Dr. McHardy and his colleagues, assisted by a most capable staff of nurses. The hospital had been built, after the most careful study of the requirements, to do the best possible for the relief of the sufferers who went there, and he was certain that no charity better deserved all the help they could give it. Sir Francis de Winton, the Treasurer, gave a great deal of time to its finances, and his connection with it was a guarantee that everything was done with the money that could be done. Hospitals generally were just now passing through a time of trial. Everyone was having to pay extra taxes; but rather than cause them to reduce their charitable expenditure the fact that the war was causing so much loss and suffering ought to make all who could not take their share in the actual fighting increase their efforts to lessen the sorrows and difficulties of the poor. The institution for which he was pleading was worked in the most economical fashion possible, and he appealed to all to back up Professor McHardy who gave his time, his services, and his abounding energy—who might in fact be said to devote his life to it.

Professor MCHARDY, the senior honorary surgeon, whose rising was the signal for loud and continued applause, replied to the toast. He said he had before been in that position but had never before felt such an access of modesty as on this occasion. Usually the chairman at these dinners, though always zealous and well-intentioned, was more or less of an amateur; but their chairman that night, though he had appealed to them to give as if he had never given himself, and though he claimed to know a good deal about Tottenham Court Road yet knew also a great deal about Gower Street. He was, as they all knew, rebuilding with no stinted hand the great hospital of University College. Like Sir Henry Irving and others who never counted the cost of a good deed, he ought to have been born a prince. He had sent for the foremost architect of the day—he would not say the best—and had gone into the matter regardless of the expense. He had spoken of £100,000, but he thought the cost would be nearer £200,000 before it was finished. Well, being thus no novice in hospital matters he had come down to St. George's Circus and had seen there how their honorary architect had buried his professional prejudices for the good of the poor patients and had acquiesced in things being done in the most practical manner possible. He had taken a great deal of trouble to make himself acquainted with all the details of the working of the institution so that he might speak that evening with personal knowledge and not upon hearsay. He, the speaker, wished all his hearers would also come down to the hospital and see what was being done. Efficiency and economy were their watchwords, and he did not know where anyone could get equal good for their eyes as was there offered to the very poorest; and if they realised what money could do at Southwark he was sure they would feel they could not possibly spend it upon a better object. In making up his subscription list for their dinner he had been greatly assisted by some who desired to give toward some memorial to the incomparable Queen-woman whose death they had all had to deplore other that the official scheme for statuary and so forth. The question of the rating of hospitals had been much discussed, but he thought there was something worse than being rated, and that was to be subjected to the authority of any centralising municipal or elected body. Theirs was a little institution, but it was doing a great work. In conclusion, the speaker paid a warm tribute to the work of his colleagues on the staff.

The SECRETARY, Mr. Edwin Euston, announced that the subscription lists totalled £1,930, that of the Chairman being £847, while Professor McHardy's was £1,001, including 700 guineas from a lady who desired in this way to raise a memorial to the late Queen.

The other toasts were "The Guests," "The Staff," and "The Chairman.'

### ROYAL FREE HOSPITAL.

THE EARL OF DARTMOUTH presided at the annual festival dinner of the Royal Free Hospital on Monday, 15th inst., at the Hôtel Cecil. After the customary loyal toasts, the Chairman proposed "Prosperity to the Royal Free Hospital." He always asked himself on occasions like this, he said, whether the institution needed support, and whether it deserved it, and with regard to the Royal Free Hospital he could answer the question indubitably. He would remind those present that the great lady who was so long a patron of the hospital had passed away. He was glad to be able to announce that the King had consented to become a patron. There was no more pathetic story than that which gave rise to this institution—the story of Dr. Marsden—which was, he thought, too well known to those present to require repetition. Monuments were of various kinds, but he did not think the founder could have a better one than this hospital. He was bound to admit that it had difficulties: one was the enormous increase of population. At the same time there was a great deal of what he might call sentimentalism in charity: new needs appealed to the public sympathy, and the old and respectable objects were liable to be forgotten. His own opinion was that the amount available was limited in extent, and that some institutions were bound to suffer when the demand was great. Everyone had demands of various kinds, and it was very difficult to know how best to respond to them. Personally he was very glad when the Prince of Wales's Fund was started, because it gave an opportunity of contributing to general hospital funds; but it was not the intention of that fund that special ones should be neglected. He was glad to say that the Royal Free Hospital had received a very generous contribution from the Prince of Wales's Fund. The financial position was a very important point, and he understood that something like £7,000 was required to complete the building. He wished to remind his audience of the great value of the institution to those for whom it was started. He noticed in the report that a very careful inquiry was made before the admission of patients: of this he highly approved, for many people who strongly resented any other form of charity were only too ready to avail themselves of medical advice at the hospitals; such persons could not realise the harm they were doing to others. Last year the total income had gone down, and that, with the continued increase of population to which he had alluded, ought not to be the case. He wished all those present would visit the hospital; they would find there a more eloquent appeal than any he could utter. "Let us," the president concluded, "do what we can to follow the example of the founder, and put this hospital on a firm foundation."

Mr. CHARLES BURT, treasurer of the hospital, who replied, said that since its foundation the hospital had benefited $2\frac{1}{2}$ millions of persons; it was a magnificent record; the present average was 100 per day. Their policy was strict economy with regard to expenditure, and the hospital had always paid its way. It had never been in debt, nor had they ever mortgaged any of their property. He strongly opposed being in debt; £40,000 had been spent on permanent building improvements, and this had been paid—not a shilling was owing. The last improvement was the accommodation for the nurses, of whom there were now fifty. The great want was a new kitchen, the present one being old and inadequate; and lifts were also greatly needed. He hoped somebody—English or American—would give them the money to provide them. Gray's Inn Road was not a rich district, and they had no millionaires there.

The Hon. JOSEPH H. CHOATE proposed "The London (Royal Free Hospital) School of Medicine for Women." "The Lord will provide," he said, "for such institutions as this, but He will be more likely to do so if they have a chairman and treasurer who state what they want." His Excellency then recalled the details of the life and work of Dr. Elizabeth Blackwell, whom he claimed as at least in some respects an American woman. It was she who set the ball rolling, and she had applied all her influence and force to it for more than fifty years. The history of the school read like a romance; the cause lay in the hearts of some half-dozen brave and devoted women whose efforts were at last successful when the doors of the Royal Free Hospital were thrown open to them, and they were admitted to full clinical courses. Since then the record had been one of continual progress. It had been said that if there was one woman who with serious purpose really desired to study medicine with the intention of making it the profession of her life, the case was entirely made out. The experience of the past 25 years had shown that there was a real need for women as doctors; the cause was bound to succeed, for it was founded on right and justice.

Mrs. GARRETT ANDERSON, M.D., dean of the school, said:—I have been thinking for many years of the debt which we Englishwomen owe to American women. They inspired Elizabeth Blackwell. But it is not so much for what they have done in freeing women as for the magnificent way in which they conducted themselves; for their extraordinary dignity in carrying on this campaign. If they had been less dignified our cause might have been wrecked. We had three objections to overcome; first, people doubted whether women could study medicine; secondly, whether they would have the courage to practise; and, thirdly, whether the women of England would care to have them. These questions, she said, had all been settled, and it had been, on the whole, rather a good thing that the choice of examining bodies was so limitted; they had to go to the highest, and the largest number had chosen the London University. Twenty-four students passed in November last. They owed a very great deal to the New Hospital for Women, where there were opportunities of seeing extremely difficult surgical operations performed. With regard to the admission of patients, the difficulty there was to pick out the most suitable ones, and if the hospital were three times the size it would be always full. Its success was largely due to the teaching the women received at the Royal Free both in lectures and in practical work in the wards. A great deal of the teaching was done in the laboratories. First-rate laboratories were an absolute necessity. There was a debt on the school, and they had to compete with very highly endowed schools; if they were to succeed they could not raise their charges very much. She had promises of endowments for anatomy and physiology, and she greatly desired to have one for chemistry. It might be said, "Is this necessary?" It was necessary to have a few women prepared to deal with those women who wished to consult them, and there was, besides, a great deal to be said in favour of women who earned their own living. She believed that the effects on the women of the future of a serious study of medicine would be very far-reaching. Knowledge was needed as well as sympathy, and if only women learnt their need of knowledge in every form a great end would be gained.

"The Medical and Surgical Staff of the Hospital" was proposed by Mr. H. J. ALLCROFT, and responded to by Dr. SAMUEL WEST and Mr. JAMES BERRY.

Towards the close of the evening the SECRETARY announced that about £4,300 had been received or promised; there had also been a donation of £1,000; and Mrs. Garrett Anderson's Students' List amounted to from £100 to £150: this was for the endowment of three "Students' Beds."

### HOSPITAL FOR CONSUMPTION AND DISEASES OF THE CHEST.

A SPECIAL meeting of governors and subscribers of the Hospital for Consumption and Diseases of the Chest, Brompton, was held on the 11th instant, Lord DERBY, the president, taking the chair. The meeting—the first one called for a special purpose for at least 40 years—was for the furtherance of the movement for the establishment of a country branch where the "Open-Air" treatment for tuberculosis can be put in practice. In opening the proceedings the CHAIRMAN said that in 1897 proposals for erecting a branch hospital in the country were put forward—though the idea was not even then by any means a new one—but at that time the circumstances were not propitious, and there did not appear to be any general desire on the part of the public to render pecuniary assistance. The project, though never allowed to drop altogether, remained in a state of suspended animation. In March, 1899, a sub-committee was appointed to ascertain what sites were procurable within a reasonable distance of London, with power to take such steps as might seem necessary to provide a branch hospital to that at Brompton, so that convalescents might enjoy purer air than was obtainable in London. Some of those present were old enough to remember when that hospital was comparatively out in the fields. But it had now been built round and enclosed by miles of streets and by masses of bricks and mortar in all directions. It was only within the last few years that the subject of the open-air treatment of tuberculosis had come to the front in this country. The Germans had been before us in this matter. That hospital had certainly led the way in the treatment of the peculiar disease it was founded to combat, and now, owing to the greater knowledge science had revealed, it was desirable they should not be satisfied with remaining as they were, but should endeavour further to adopt the latest known means for the prevention and treatment of this dreadful disease. The sub-committee had made a report in pursuance of which the governors and subscribers had been called together that day, and they hoped that, by bringing their scheme in its present state before the public, they would receive the generous support for which they were appealing. They were not going in for an elaborate architectural design. Mr. Hall, the architect, had prepared a plan from which it would be seen that he had, as far as possible, and at a comparatively moderate cost, endeavoured to provide all the arrangements required for outdoor treatment. The estimate was a very moderate one indeed. It was not to be supposed for a moment that the sanatorium would be a distinct establishment. The committee were practically unanimous in the desire to carry on the hospital under the general rules of management which had hitherto proved so efficient. The branch would be an accessory to it, working through this hospital, and not separately from it. He felt satisfied they were going forward in a good work, and that they had the sympathy and ought to have the help of the entire community.

Major-General EATON said that for many years they had been sending patients to various convalescent homes, and the committee had come to the conclusion that they ought now to have one of their own. That was the original design in 1897, but the response to the appeal then made had not been great, only some £2,000 having been subscribed. Then the open-air treatment began to attract public attention, and it became necessary to take a prominent part in it. The sub-committee appointed inspected a number of sites, and ultimately that on Chobham Ridges, between Bagshot and Frimley, was decided on, and they had acquired 20 acres freehold, at a cost of £4,000. They hoped in less than two years to be in possession of the new premises. Their object in calling the meeting was, of course, to ask the governors and their friends generally to do all in their power to raise the necessary funds. The approximate cost of the buildings would amount to £40,000, and he could not imagine that when the country knew what the hospital was doing in this matter it would leave them long without the necessary funds. They looked to the City Companies to help them, and to the rest of the country to follow so good a lead.

Mr. G. ST. CROIX ROSE said he had a peculiar interest in the advance now proposed in that he might claim that his father had originated that hospital. It had become the principal hospital in the country, indeed in the world, for consumption, and he recommended most strongly the carrying out of the scheme. But he was anxious to correct the impression that the hospital itself was going in any way to lessen its usefulness. Cases must continue to come there for diagnosis and to be sorted and classified, for it must not be forgotten that they treated not only consumption but all forms of diseases of the chest. He urged all present to get their friends to give them all the support they could and recommended that the committee should go ahead with the scheme and trust to Providence for the money,

Dr. POLLOCK, Senior Consulting Physician, said that as the oldest member of the medical staff he could remember the early days of the institution, and could assure them that the movement now in progress was in keeping with their best traditions. Medical science had shown them new facts and called forth new theories. They knew now that it was possible for tuberculosis to be communicated from person to person, and also by means of meat and milk. And if they were able to benefit sufferers so as to get, for instance, the father of a family back to his work, they were benefiting not only their patients but the whole community.

Dr. SYMES THOMPSON and Sir DOUGLAS POWELL, consulting physicians to the hospital, also advocated the proposed step.

Dr. THEODORE WILLIAMS said that he and his colleagues on the committee had gone very thoroughly into the details of this matter. They had visited many places on the Continent, and their architect had done so also, with the result that the plans now submitted, if not actually perfect, were adapted to their needs. As regards the site, it was a very beautiful one; the soil was excellent; the water supply was good; and in the matter of isolation they had 40,000 acres belonging to the War Office on one side, and Frimley Common on the other. The view was magnificent—clear away to the Hog's Back. And in addition they were well sheltered by a grove of fir trees. It would be a great advantage to the parent hospital to have this branch which would relieve the pressure on their beds very materially. If they left this matter undone they would be what they had never been before—behind the age. In fact they would have carried out the scheme before this had it not been for lack of funds.

Dr. ACLAND, Dr. MAGUIRE, and Dr. HORTON SMITH also advocated the scheme, the last-named mentioning that they frequently had cases in the out-patient department which might be saved if sent at once to a country branch, but in which the condition after six weeks or two months' waiting was so much worse as to make them hopeless as regarded cure.

Mr. E. T. HALL, the architect, then explained his plans, after which LORD DERBY announced that he had received a promise of £1,000 from one who wished to remain anonymous.

A vote of thanks to the Chairman brought the proceedings to a close.

